# Efficacy and challenges of CRPP for Jakob type III lateral humeral condylar fractures: an age-stratified retrospective cohort study of 128 patients

**DOI:** 10.3389/fped.2026.1795857

**Published:** 2026-04-10

**Authors:** Weizhe Shi, Yanhan Liu, Yi Qiu, Meiying Ruan, Honghong Lin, Jianping Wu, Xuemei Lin, Zhe Kang, Jianqun Wang, Zhong Wei, Yiqiang Li, Hongwen Xu

**Affiliations:** 1 Zunyi Medical University, Zunyi, China; 2 Department of Pediatric Orthopaedics, GuangZhou Women and Children’s Medical Center, GuangZhou Medical University, GuangZhou, China

**Keywords:** age-stratified analysis, closed reduction and percutaneous pinning, lateral humeral condylar fractures, ORIF (open reduction and internal fixation), retrospective cohort study

## Abstract

**Objective:**

Jakob Type III lateral humeral condylar fractures are common inarticulate fractures in pediatric population, and inadequate management may result in severe complications. This study aimed to evaluate the age-specific efficacy of closed reduction and percutaneous pinning (CRPP) in pediatric patients.

**Methods:**

A retrospective analysis was performed on 128 pediatric patients with Jakob III type lateral humeral condylar fractures who underwent CRPP at our institution from 2018 to 2024. Patients were stratified into three age groups: toddler group, preschool group, and school-age group. Intraoperative conversion to open reduction occurred in 11 cases in the toddler group and 1 case in the preschool group. All patients were followed up for at least 12 months. Outcome measures included surgical duration, intraoperative fluoroscopy time, 1-year MEPS, intraoperative conversion rate, primary reduction success rate, carrying angle, complication rate, and reoperation rate.

**Results:**

Comparative analysis of the three groups revealed the following findings: The toddler group had significantly longer surgical duration (98.0 ± 35.25 min) and fluoroscopy time (80.9 ± 32.4 s) than preschool and school-age groups (all *P* < 0.05). All groups achieved excellent 1-year MEPS (>90) with no intergroup difference (*P* > 0.05). Toddlers showed higher conversion rate and lower primary reduction success (both *P* < 0.05). Postoperative carrying angle did not differ among groups. Within toddlers, no differences were found between CRPP and open reduction patients in MEPS, complications, reoperations, or angle changes.

**Conclusion:**

CRPP achieves favorable outcomes for pediatric patients with Jakob III lateral humeral condylar fractures across all age groups. However, the toddler group (0–3 years) exhibits unique therapeutic challenges: immature ossification complicates intraoperative fluoroscopic assessment, necessitating more frequent intraoperative strategy adjustments. Although the toddler group experiences higher CRPP difficulty and failure rates, timely conversion to open reduction and internal fixation (ORIF) as a salvage procedure yields comparable short-term functional outcomes to successful CRPP cases. These findings support age-based individualized surgical planning and flexible intraoperative adjustment strategies.

## Introduction

Fractures of the lateral humeral condyle in children are a common type of fracture, among which Jakob Type III fractures pose significant therapeutic challenges due to their complete displacement and intra-articular involvement (1). Inadequate management of such fractures may result in severe complications including nonunion, malunion, cubitus varus, and joint stiffness, which not only impair elbow function and quality of life in affected children but also impose long-term financial burdens on families (2). Currently, closed reduction and percutaneous pinning (CRPP) has become the mainstream treatment for humeral lateral condyle fractures due to its minimally invasive advantages, supported by accumulating evidence (3, 4). However, despite widespread adoption, limited research exists regarding age-specific efficacy of this technique, particularly in the toddler group (0–3 years) with immature skeletal development. This study employed the retrospective analysis to conduct age-stratified analysis of 128 children with Type III lateral humeral condyle fractures treated with closed reduction and percutaneous Kirschner wire fixation. Patients were stratified into three age groups: toddler group (0–3 years), preschool group (4–6 years), and school-age group (≥7 years). This design utilizes real-world clinical data to evaluate age-related impacts on treatment outcomes. The primary objective was to compare 1-year postoperative elbow function recovery among groups using the internationally validated Mayo Elbow Performance Score (MEPS) (5). Secondary objectives included assessment of differences in operative duration, intraoperative radiation exposure, conversion rate to open reduction, primary reduction success rate, complication rate, reoperation rate, and final follow-up x-ray elevation angle.

This study aims to clarify whether age represents a key variable influencing treatment decisions and outcomes for Jakob Type III humeral lateral condylar fractures. Findings are expected to provide age-stratified treatment references for clinicians, particularly regarding the unique pediatric population, facilitating balanced assessment of minimally invasive surgery benefits and risks, optimizing treatment strategies, and ultimately improving overall prognosis across all age groups.

## Materials and methods

The retrospective analysis was approved by the Medical Ethics Committee of Guangzhou Maternal and Child Health Hospital and strictly complied with the ethical principles of the Declaration of Helsinki of the World Medical Association. This study has been registered on the clinical trial registry website (http://www.clinicaltrials.gov) with the registration number NCT04640727 (registered on January 20, 2022).

All research data were derived from routine clinical records, and the personal identifiable information of all patients was anonymized to protect their privacy. Written informed consent from patients was waived with the approval of the aforementioned ethics committee, as this was a retrospective study with no potential harm to patients.

All authors (WS, YL, YQ, MR, HL, JW, XL, ZK, JW) and corresponding authors (YL, HX) confirm that there are no ethical disputes related to this study and voluntarily assume full responsibility for the authenticity of this statement.

Through reviewing medical records, we screened pediatric and adolescent patients with humeral lateral condylar fractures who visited the orthopedic department of our hospital between January 2018 and December 2024. All patients were consecutively enrolled, and all fractures were treated by the same surgical team at a single institution. The mean follow-up duration was 16.3 months (range: 12.3–41.9 months). Patients diagnosed with Jakob Type III lateral condylar fractures were categorized by age into toddler group (0–3 years), preschool group (4–6 years), and school-age group (≥7 years). CRPP was the preferred treatment modality. Collected follow-up data included: gender, age of injury, affected side, presence of comorbidities or neurovascular complications, and whether the fracture was closed or open. Imaging examinations were performed using the Arcadis Varic image enhancement system (Siemens, Munich, Germany). This study employed a pulsed scanning method (one pulse per second) with automatic optimization of dose, brightness, and contrast during image acquisition. Inclusion criteria were as follows: (1) Confirmed as Jakob Type III lateral condylar fractures; (2) Standard preoperative anteroposterior (AP) and lateral x-ray films of the elbow; (3) Follow-up duration >12 months; (4) Complete clinical and imaging data. The exclusion criteria were as follows: (1) patients with multiple injuries complicated by other fractures; (2) open fractures or pathological fractures; (3) follow-up time <12 months; (4) patients treated at other institutions; (5) incomplete imaging data. Each fracture was identified, assessed, and classified according to the JFC system using anteroposterior and lateral radiographs of the elbow joint, and the presence of other concurrent bone lesions was examined.

Closed reduction and percutaneous pinning (CRPP) Surgery is performed with the patient supine and without the use of a tourniquet. The following steps are performed by the surgeon:

The patient is placed in a supine position with the affected limb abducted and positioned within the fluoroscopy field, while comprehensive radiation protection measures are implemented for the child. We emphasize the importance of employing ALARA (As Low As Reasonably Achievable) principles, including minimizing exposure time, using pulsed fluoroscopy, and employing appropriate shielding, especially in this younger, more radiosensitive population.

Evaluate the amount of displacement and rotation of the fragment under fluoroscopy on AP and lateral images.

Insert percutaneously a 2.5-mm K-wire in the articular cavity to evacuate the haematoma, reduce the swelling of the elbow, and facilitate palpation of the fragment.

Convert the type 3 LCF into a type 2 fracture. The surgeon must apply traction along the axis of the upper extremity while applying varus force at the level of the elbow to create a space for the reduction of the fractured fragment. At this point, gradual compression on the distal fragment both medially and anteriorly by the surgeon's thumb should be applied to achieve reduction. If the manoeuvre does not allow repositioning of the rotated fragment, a 2-mm Kirschner (K)-wire can be inserted percutaneously into the fragment and used as a joystick to help reposition the rotated fragment.

The type 2 LCF is converted into a type 1 fracture by further compression of the distal fragment, with the elbow in extension. Once the fracture gap is <2 mm on the AP view, the fragment can be temporarily fixed with a 1.6 mm smooth K-wire.

Check the reduction on the lateral view, and definitively stabilize the fracture by 2 K-wires. The reduction should be evaluated on lateral view; if the fracture gap is >2 mm, the surgeon, now holding the elbow in flexion, applies pressure to the distal fragment in the postero-anterior direction and withdraws, at the same time, the 1.6 mm K-wire to allow closure of the fracture line. Once sufficient inter fragmentary compression has been achieved, the 1.6-mm K-wire can be advanced again to stabilize the fracture. At this point, three 2-mm divergent K-wires can be inserted, and the 1.6-mm K-wire can be removed.

To confirm the quality of the reduction on the oblique view, if the fracture gap is >2 mm, the K-wires should be removed and compression on the fragment exerted to achieve reduction. Once satisfactory reduction is achieved, three K-wires are used to stabilize the fracture.

An arthrogram is performed to evaluate the congruency of the articular cartilage;

The elbow is immobilized in a long arm cast at 90° of flexion.

Satisfactory reduction” was defined as a residual fracture gap of ≤2 mm on intraoperative fluoroscopy, assessed primarily on anteroposterior (AP), lateral, and internal oblique views. The internal oblique view was particularly decisive for evaluating the articular surface. Arthrography was also routinely used in our series. The primary criterion for conversion to ORIF was the inability to achieve or maintain this ≤2 mm reduction via closed means after two attempts.

### Follow-up protocol

At each follow-up visit (1 month, 3 months, 12 months, and annually thereafter), anteroposterior (AP) and lateral elbow x-rays should be taken to assess the progress of fracture healing and detect potential complications such as internal fixation displacement, secondary displacement, malunion, delayed union, nonunion, infection, neurovascular injury, refracture, or joint stiffness.

### Clinical outcomes

The clinical outcomes were evaluated based on the Mayo Elbow Performance Score (MEPS) at 1 year postoperatively.

Additionally, Primary reduction success rate (defined as achievement of satisfactory reduction with three Kirschner wires, check the AP and lateral view, the fracture gap is <2 mm, and no significant abnormalities were observed on the joint angiography).

And Intraoperative conversion rate (defined as conversion to open reduction and internal fixation due to failure to achieve satisfactory reduction after standardized CRPP procedures, Including: 1. Failed attempts to convert Type 3 fracture into Type 2 fracture; 2. Inadequate reduction of Type 2 fracture in both anteroposterior and lateral views; 3. Post-reduction arthrography revealing irregular articular surfaces. Kirschner wire insertion, radiography, and arthrography.) were recorded. Complications and reoperation rate, including avascular necrosis (AVN), growth arrest, and infection, were also documented. The final follow-up included anteroposterior elbow radiography to assess the carrying angle, defined as the angle between the humeral and ulnar axes.

### Statistical analysis

Continuous data among the three groups are presented as mean ± standard deviation (SD) and compared using one-way ANOVA with *post-hoc* Bonferroni correction. Categorical data are expressed as percentages and compared using the *χ*^2^ test. Ranked data were analyzed with the Kruskal–Wallis test. All tests were two-tailed, with a significance level of *P* < 0.05, and performed using SPSS software (version 22.0; IBM Corp., Armonk, NY, USA).

## Results

**Figure 1:**
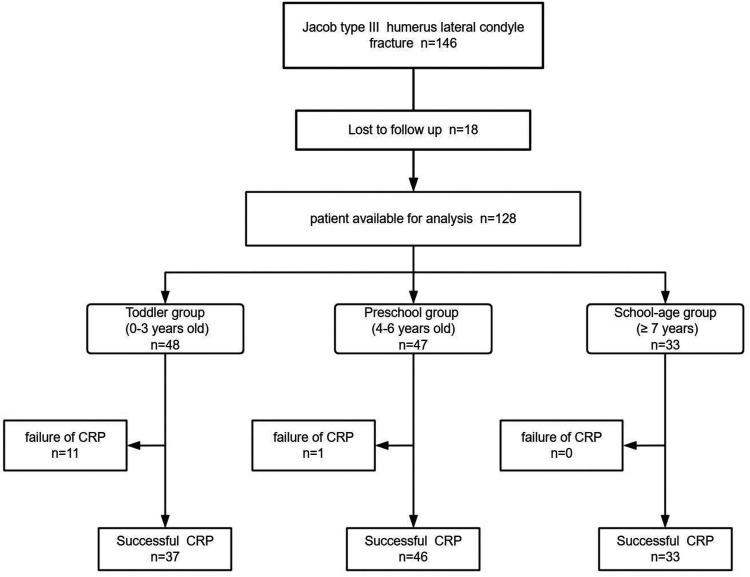
Flowchart of the patients.

This study enrolled 48 children in the toddler group, 47 in the preschool group, and 33 in the school-age group, with all patients followed up for at least 12 months (Detailed data are shown in Table 1). Among the three age groups, the number of patients successfully undergoing closed reduction surgery was 37 in the toddler group, 46 in the preschool group, and 33 in the school-age group (Detailed data are shown in Figure 1). The operative time was 98.0 ± 35.25 min in the toddler group, 66.6 ± 29.32 min in the preschool group, and 70.6 ± 23.52 min in the school-age group. The fluoroscopy time was 80.9 ± 32.4 s in the toddler group, 50.5 ± 32.02 s in the preschool group, and 34.5 ± 13.87 s in the school-age group. Comparisons among the three age groups revealed that the toddler group had significantly longer operative and fluoroscopy times compared to the school-age and preschool groups (*P* < 0.05).

**Table 1:** Baseline characteristics of patients.

Characteristic	Toddler group (*n* = 48)	Preschool group (*n* = 47)	School-age group (*n* = 33)
Age (years)	2.29 ± 0.68	4.74 ± 0.67	8.30 ± 1.42
Sex (male/female)	35,13	30,17	19,14
Side (left/right)	21,26	14,23	10,23

A comparative analysis of patients with successful closed reduction was conducted, evaluating 1-year postoperative Mayo Elbow Performance Score (MEPS), intraoperative conversion rate, primary reduction success rate, complications, and reoperation rate. The results showed 1-year MEPS scores as follows: toddler group 91.6 ± 5.22, preschool group 93.4 ± 3.96, and school-age group 95.7 ± 2.57. Primary reduction success rates were 76% in the toddler group, 89% in the preschool group, and 93% in the school-age group. Intraoperative conversion rates were 22% in the toddler group, 2% in the preschool group, and 0% in the school-age group. Reoperation rates were 4% in the toddler group, 0% in the preschool group, and 0% in the school-age group. Complications occurred in 3 cases in the toddler group, 2 cases in the preschool group, and 2 cases in the school-age group. No significant difference was observed in 1-year MEPS among groups (*P* > 0.05). The toddler group had significantly lower primary reduction success rate (*P* < 0.05) and higher intraoperative conversion rate (*P* < 0.05) compared to the other two groups. Postoperative x-ray assessment of carrying angle showed no significant differences among groups (*P* > 0.05) (Detailed data are shown in Table 2).

**Table 2:** The clinical outcome data of the three age groups were analyzed.

Assessment indicators	Toddler group (*n* = 37)	Preschool group (*n* = 46)	*P* value	Toddler group (*n* = 37)	School-age group (*n* = 33)	*P* value	Preschool group (*n* = 46)	School-age group (*n* = 33)	*P* value
Fluoroscopy time (seconds)	80.9 ± 32.4	50.5 ± 32.02	<0.05	80.9 ± 32.4	34.5 ± 13.87	>0.05	50.5 ± 32.02	34.5 ± 13.87	<0.05
OR time (minutes)	98.0 ± 35.25	66.6 ± 29.32	<0.05	98.0 ± 35.25	70.6 ± 23.52	>0.05	66.6 ± 29.32	70.6 ± 23.52	<0.05
Mayo Elbow Performance Score (MEPS)	91.6 ± 5.22	93.4 ± 3.96	>0.05	91.6 ± 5.22	95.7 ± 2.57	>0.05	93.4 ± 3.96	95.7 ± 2.57	>0.05
Primary reduction success rate	0.756756757	0.891304348	<0.05	0.756756757	0.939393939	>0.05	0.891304348	0.939393939	>0.05
Carrying angle	18.6 ± 8.32	16.2 ± 9.31	>0.05	18.6 ± 8.32	15.5 ± 9.93	>0.05	16.2 ± 9.31	15.5 ± 9.93	>0.05
Intraoperative conversion rate	22%	2%	<0.05	22%	0	<0.05	2%	0	>0.05
Reoperation rate	4%	0	>0.05	4%	0	>0.05	0	0	>0.05
Complication	3	2	>0.05	3	2	>0.05	2	2	>0.05
Non-union	0	0		0	0		0	0	
Skin infection	3	1		3	2		1	2	
Septic arthritis	0	1		0	0		1	0	
Re-fracture	0	0		0	0		0	0	
Avascular necrosis	0	0		0	0		0	0	
Growth arrest	0	0		0	0		0	0	

In the toddler group, comparison between CRPP (*n* = 37) and intraoperative converted ORIF (*n* = 11) showed no significant differences in 1-year MEPS, carrying angle, complications, or reoperation rate (*P* > 0.05) (Detailed data are shown in Table 3)

**Table 3:** Analysis of clinical outcome data between CRPP and ORIF.

Assessment indicators	Toddler group CRP *n* = 37	Toddler group ORIF *n* = 11	*P* value
Mayo Elbow Performance Score (MEPS)	91.6 ± 5.22	92.32 ± 7.32	>0.05
Carrying angle	18.6 ± 8.32	20.3 ± 7.53	>0.05
Reoperation rate	2	0	>0.05
Complication	1	2	
Non-union	0	0	
Skin infection	1	2	
Septic arthritis	0	0	
Re-fracture	0	0	
Avascular necrosis	0	0	
Growth arrest	0	0	

## Conclusion

Lateral humeral condyle fractures are the second most common type of elbow fracture in children, accounting for 12% to 20% of pediatric upper extremity fractures (1). Children aged 4 to 10 years are at the highest risk, with the peak incidence occurring at 6 years old. The most common mechanism of injury is a fall onto an outstretched hand with elbow extension and forearm supination (6); other mechanisms include direct elbow trauma and valgus compression (7). As a common intra-articular elbow injury in children, improper treatment of lateral humeral condyle fractures may lead to elbow deformity, functional impairment, and long-term complications, significantly affecting the quality of life of affected children (8, 9). Although several classification systems for lateral condyle fractures exist, the Milch (10) and Jakob (11) classifications are most widely used in clinical practice. The Jakob classification categorizes fractures based on displacement and rotation of the lateral condyle fragment into three stages: Stage I with <2 mm displacement indicating intact cartilaginous joint; Stage II with 2–4 mm displacement without fragment rotation; and Stage III with >4 mm displacement and fragment rotation (11). This classification demonstrates good consistency between preoperative radiographs and intraoperative findings, with Jakob type III fractures presenting greater treatment challenges due to complete fragment displacement. Currently, closed reduction and percutaneous pinning (CRPP) has gained increasing support due to its minimally invasive advantages (12). However, patient age, particularly in young children, may significantly impact CRPP success rates and technical difficulty due to immature skeletal development and high cartilaginous component. Therefore, systematic evaluation of age-stratified treatment outcomes is crucial for optimizing individualized treatment strategies.

This retrospective analysis with prospectively enrolled patients aimed to compare the primary reduction success rate, intraoperative decision modification rate, operative time, and radiation exposure duration of CRPP in children with Jakob III lateral humeral condyle fractures across different age groups (toddler, preschool, and school-age). The study also evaluated 1-year postoperative elbow function using the Mayo Elbow Performance Score (MEPS) and complication rates, with special comparison between successfully reduced and failed CRPP cases in the toddler group to assess the efficacy of open surgery as a salvage procedure. These findings will provide important decision support for clinicians treating pediatric lateral humeral condyle fractures, particularly in high-risk toddler patients. The main results indicate significant age-related differences in CRPP applicability for pediatric Jakob III lateral humeral condyle fractures. Despite an overall high success rate (90.6%), the toddler group (0–3 years) had significantly lower reduction success rates compared to preschool (4–6 years) and school-age (≥7 years) groups, with a higher conversion rate to open reduction and internal fixation (22.9%). This emphasizes the importance of age consideration in surgical decision-making, especially for toddler patients. Skeletal characteristics of young children, such as small fracture fragments, high cartilaginous content, and poor reduction stability, may increase surgical difficulty and decrease success rates (13). Therefore, thorough preoperative assessment and preparation for potential intraoperative adjustments are essential to improve overall treatment outcomes.

Potential complications following pediatric lateral condyle fractures include lateral condyle overgrowth, cubitus varus/valgus, fish tail deformity, osteonecrosis, nerve injury, physeal growth arrest, and osteoarticular dysplasia (14). This study found that no statistically significant differences in 1-year postoperative MEPS were observed between age groups in patients successfully treated with CRPP, indicating that satisfactory long-term functional recovery can be achieved with adequate reduction and fixation regardless of age. Comparison between toddlers with successful CRPP and those converted to open reduction showed no significant differences in 1-year MEPS or complication rates. This finding supports the safety and efficacy of flexible intraoperative strategy adjustment, suggesting that timely conversion to ORIF does not compromise final functional outcomes when encountering intraoperative difficulties. Our study not only confirmed age-related technical challenges but also validated the long-term functional equivalence of the stepwise strategy of “CRPP first, prompt conversion to ORIF if failed” in the toddler population, providing relevant evidence-based support for clinical decision-making. Additionally, operative time and radiation exposure were significantly higher in the toddler group, quantifying the technical challenges of surgery in this age group. Nevertheless, complication rates showed no significant intergroup differences, which may be attributed to experienced surgical teams, strict operative indications, and standardized follow-up protocols. Therefore, special attention should be paid to intraoperative monitoring and radiation protection when operating on toddler patients to ensure surgical safety and efficacy. Overall, this study emphasizes the importance of individualized treatment protocols for pediatric fractures across different age groups and provides reference for clinical decision-making.

This study has several limitations. First, as a single-center retrospective study, despite prospectively enrolled patients to standardize data collection, potential selection bias may still exist, at the same time, this study did not analyze learning time trends and surgical experience as covariates, confounding factors may still exist. Second, the sample size is significantly limited, particularly the small subgroup of toddlers experiencing initial CRPP failure who were converted to ORIF; this subgroup included only 11 patients. Consequently, the ORIF cohort primarily represents a salvage procedure following failed CRPP, thereby limiting direct comparative conclusions between CRPP and ORIF as primary planned treatment interventions.

Additionally, the follow-up period remains notably abbreviated; although sufficient for assessing union and short-term functional outcomes, it is insufficiently prolonged to reliably evaluate potential long-term complications such as fishtail deformity, growth disturbance, or angular changes. These critical outcomes warrant rigorous investigation in dedicated long-term follow-up studies. Pediatric bones possess remarkable remodeling capacity, yet are not immune to delayed complications, such as the well-documented ischemic necrosis of the humeral trochlea following lateral humeral condyle fractures (15), making longer-term follow-up crucial. Future studies should expand sample sizes, conduct multicenter collaborations, and extend follow-up until skeletal maturity to further optimize treatment decision-making processes for young children and explore imaging or clinical features that can preoperatively predict CRPP failure risk, thereby achieving more precise individualized treatment.

In conclusion, this study systematically evaluated the efficacy of CRPP in treating Jakob III lateral humeral condyle fractures across different pediatric age groups. Despite the higher conversion rate to open surgery in the toddler group, all age groups achieved favorable short-term functional outcomes with satisfactory reduction. The results emphasize the importance of age-based individualized surgical planning and confirm the feasibility of flexible intraoperative strategy adjustments. Future research should focus on expanding sample sizes and conducting long-term follow-up to further clarify the long-term effects of different treatment strategies on skeletal development and joint health.

## Data Availability

The original contributions presented in the study are included in the article/supplementary material, further inquiries can be directed to the corresponding authors.
